# Clinical Characteristics of 1378 Inpatients with Spinal Tuberculosis in General Hospitals in South-Central China

**DOI:** 10.1155/2019/9765253

**Published:** 2019-03-03

**Authors:** Zheng Liu, Jun Wang, Gong-Zhou Chen, Wei-Wei Li, Yun-Qi Wu, Xiao Xiao, Yi-Lu Zhang, Yan Yang, Wen-Kai Hu, Zhi-Cheng Sun, Xi-Yang Wang

**Affiliations:** ^1^Department of Spine Surgery, Xiangya Hospital, Central South University, 87#Xiangya Road, Changsha 410008, Hunan, China; ^2^Hunan Engineering Laboratory of Advanced Artificial Osteo-materials, Xiangya Hospital, Central South University, 87#Xiangya Road, Changsha 410008, Hunan, China

## Abstract

In this retrospective study, charts of inpatients with spinal tuberculosis (STB) treated in large-scale general hospitals in Changsha, Hunan, China, between 2007 and 2016 were reviewed to investigate their clinical characteristics. Demographic, epidemiological and clinical features, imaging findings, treatment methods, and prognosis were summarized and analyzed. There were 1378 patients, 805 males and 573 females, with a mean age of 43.7 years. The mean interval between symptom onset and diagnosis was 16.0 months (range 15 days–240 months). The incidence of back pain, radicular pain and symptoms of systemic toxicity was 92.5%, 40.1%, and 32.1%, respectively. The rate of neurological impairment was 49.9 %. STB was present in two or more vertebrae in 91.1% of patients, with two adjacent vertebrae being involved in 67.9% of them. The lumbar segment (38.2%) was the most frequently affected, followed by the thoracic spine (35.7%). The sacrococcygeal area was the least frequently involved (0.8%). Abscesses were detected in 65.5% of patients. One thousand patients (72.6%) were managed with surgery and 378 (27.4%) with anti-TB drugs only. Cure was achieved in 1215 patients (88.2%), whereas 49 (3.5 %) had relapses. Concomitant pulmonary TB (PTB) was diagnosed in 366 patients (26.6%) and 63 (4.6%) had concomitant diabetes. Compared with the previous five years, the number of older patients, urban patients, and medical staff with STB had increased by 6.1%, 5.2%, and 1.3%, respectively in the five years studied. STB remains a severe public health problem that cannot be ignored. Most of the patients ignored early symptoms and therefore received untimely treatment. Thus, surveillance for and treatment of STB in South-central China requires strengthening. In addition to the current China-wide database of patients with PTB, a China-wide database of patients with STB should also be set up.

## 1. Introduction

According to a 2018 report of the World Health Organization (WHO) [1], although the incidence and mortality of tuberculosis (TB) in China have been declining slowly in recent years, China still had the second highest burden of TB in the world, with about 889,000 new cases of TB and 388,000 deaths in 2017, second only to India. Thus, TB is still a major problem, endangering the health of Chinese people.

Spinal tuberculosis (STB), which accounts for half of bone and joint TB and is the most common form of extrapulmonary TB, often leads to irreversible neurological injury, including paralysis, resulting in serious social and economic problems [2]. STB is a chronic disease that is slowly progressive, and diagnosing it is relatively difficult [3].

Progression of STB may cause spinal deformity and neurological injury, including paralysis, which seriously affect the quality of lives of affected patients and are difficult to cure by chemotherapy drugs. Anti-TB drug chemotherapy is the major means of treating STB and is the fundamental means of curing STB, with surgery being an adjuvant means of treatment [4, 5].

Clinical data concerning TB differ according to differences in geographical locations, life style, level of economic development, level of available medical services, and other factors. In Hunan province, located in south-central China, there is a high incidence (83.0/10 million) and high number of reported cases (55,919 cases) of pulmonary tuberculosis (PTB), resulting in this province ranking in the top eight and three provinces of China, respectively (data obtained from the notifiable PTB report of the Chinese Infectious Disease Report System [IDRS]). A number of epidemiological studies of PTB in Hunan have been published, but none concerning STB. This is also true for throughout China.

In this study, clinical data of inpatients with STB in Changsha, Hunan, from 2007 to 2016, including general information, symptoms of TB, diagnosis, treatment, and prognosis, were collected and analyzed to help in the prevention, diagnosis, treatment, and prognosis of STB in Hunan province.

## 2. Materials and Methods

### 2.1. Patients Survey

In this retrospective study, the medical records of inpatients admitted for STB to several large comprehensive hospitals in Changsha, Hunan, China, from January 2007 to December 2016 were collected and reviewed.

Diagnosis of STB was established by clinical, laboratory, and imaging investigations supplemented by pathological examination of biopsy specimens. The diagnosis was based on the following criteria: (1) clinical symptoms and signs such as backache, spinal deformity, neurological deficit (numbness, weakness, dysesthesia, dyskinesia, and paralysis), systemic toxicity symptoms (STS) of TB, including low-grade fever, night sweats and emaciation; (2) computed tomography (CT) and magnetic resonance imaging (MRI) manifestations, such as destruction of vertebral bone, intervertebral space involvement, kyphosis, paravertebral abscess formation, and dural sac compression; (3) laboratory findings including high erythrocyte sedimentation rate (ESR) and C-reactive protein (CRP) concentration, positive T-SPOT, TB antibody (IgG and IgM), and tuberculin test; (4) diagnostic anti-TB treatment was effective when it was difficult to otherwise make a definite diagnosis; (5) pathological evidence of TB, such as typical TB with caseous necrosis microscopically, was regarded as the golden standard of diagnosis.

All patients who were diagnosed with STB were routinely treated with the HREZ anti-TB chemotherapy regimen for 12–18 months, namely, rifampicin (450 mg/day), isoniazid (300 mg/day), ethambutol (750 mg/day), and pyrazinamide (750 mg/day). Kidney function and liver function were monitored during anti-TB chemotherapy.

Indications for surgery for STB were as follows: (1) progressive or severe neurological damage, (2) bone destruction of various degrees with severe or developing spinal deformity, (3) persistent back pain due to spinal instability, and (4) poor response to medical treatment.

### 2.2. Clinical Data

On hospital admission, personal characteristics, clinical manifestations, imaging findings, comorbidities, treatment, and prognostic indicators were collected and analyzed. Patients with missing personal, clinical, or examination data were excluded from the study. Detailed clinical data, including sex, age, place of residence (rural versus urban), occupation, local pain, radicular pain, STS, abscess, neurological injury, involved vertebrae, locations of lesions, concomitant PTB or diabetes, and treatment, were summarized. The American Spinal Injury Association (ASIA) impairment scale was applied to score neurological function. Correlations between variables and years of study were analyzed.

Patients with STB were divided into two groups according to the year of admission, Group A from January 2007 to December 2011 and Group B from January 2012 to December 2016, and variables were assessed comparing the two groups. Trends in changes of each variable were summarized. Because STB is considered to originate from PTB and diabetes is a common comorbidity of STB, correlations between PTB, diabetes, and STB were analyzed.

Follow-up information was obtained by telephone, through e-mail, and during return visits to the outpatient department. “Cure” was defined as follows: no recurrence of TB lesions within 2 years after treatment, maintenance of ESR within the normal range, identification of bone union in lesions by radiographic examination, and disappearance of clinical symptoms for 3 months.[6]

### 2.3. Statistical Analysis

Statistical analysis of the experimental data was performed with the *χ*^2^ test using SPSS 24.0 software (SPSS, Chicago, IL, USA). P < 0.05 was considered to denote significant differences.

## 3. Results

### 3.1. Demographic, Epidemiological, and Personal Characteristics

During the years of this study, there were 1378 eligible inpatients with STB.  Table 1 shows the demographic, epidemiological, and personal characteristics of the study patients. During this 10-year period, the incidence of STB was relatively stable (116–170 new cases per year). The study cohort comprised 805 (58.4%) male and 573 (41.6%) female patients. In the 10 years of the study, there were always more male than female patients. Additionally, there was no significant difference in the ratio of male to female during these 10 years (P > 0.05).

**Figure 1:**
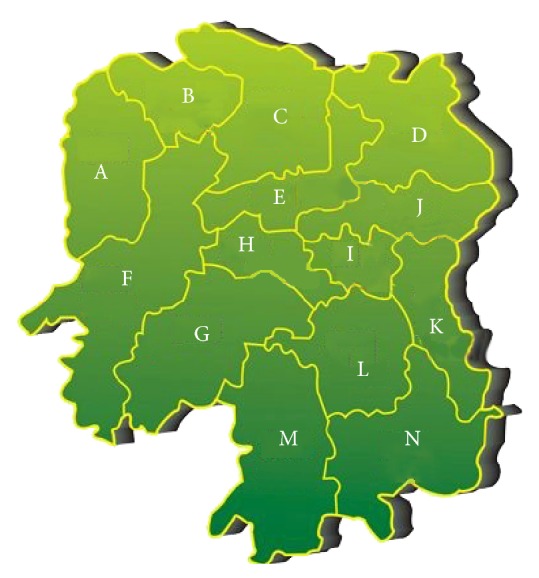
The distribution of patients with STB in Hunan province is shown: 214 (15.53%) were from Changsha (J), 161 (11.68%) from Shaoyang (G), 130 (9.43%) from Yueyang (D), 128 (9.29%) from Yiyang (E), 121 (8.78%) from Loudi (H), 111 (8.06%) from Yongzhou (M), 92 (6.68%) from Hengyang (L), 89 (6.46%) from Zhuzhou (K), 82 (5.95%) from Changde (C), 51(3.70%) from Xiangtan (I), 42 (3.05%) from Zhangjiajie (B), 30 (2.18%) from Chenzhou (N), 41 (2.98%) from Huaihua (F), and 26 (1.89%) from Xiangxi (A).

The age range was 1–88 years, with the mean age being 43.7 years. Specifically, 561 patients (40.7%) were aged from 18 to 45 years, followed by the 46–60 years-old (27.2%). As to place of residence, urban patients accounted for 29.2% (375/1378) and rural patients 72.8% (1003/1378) of the cohort. The mean interval between symptom onset and diagnosis in the hospital was 16.0 months (range 15 days–240 months), this interval being a mean of 22.0 months in rural patients and 9.6 months in urban patients; this difference is significant (P<0.05). In addition, the distribution of patients with STB in various cities of Hunan province is shown in Figure 1 (Changsha, 15.53%; Shaoyang, 11.68%; Yueyang, 9.43%; Yiyang, 9.29%; Loudi, 8.78%; Yongzhou, 8.06%; Hengyang, 6.68%; Zhuzhou, 6.46%; Changde, 5.95%; Xiangtan, 3.70%; Zhangjiajie, 3.05%; Chenzhou, 2.18%; Huaihua, 2.98%; Xiangxi, 1.89%). The remaining 4.35% were from Jiangxi and Guizhou province, both adjoining Hunan province.

As to distribution of occupations, there were 659 farmers (47.8%), 259 manual workers (18.8%), 128 students (9.3%), and 332 with other occupations (24.1%). The correlation between occupations and years `was analyzed. Distribution of occupation did not differ significantly over time (P > 0.05).

### 3.2. Clinical Presentation

As to the clinical presentation of patients with STB (Table 2), 92.5% presented with back pain and the remaining 7.5% with spinal deformity or numbness in the limbs without back pain. The incidences of radicular pain and STS in patients with STB were 40.1% and 32.1%, respectively. Analysis of correlations between back pain and radicular pain and year of study showed no significant change in the incidence of these symptoms over the 10 years (P > 0.05). As to distribution of severity of neurological injury, 21 (1.5%) patients had ASIA Grade A deficits, 38 (2.8%) Grade B, 111 (8.1%) Grade C, 517 (37.5%) Grade D, and 691 (50.1%) Grade E.

### 3.3. Imaging Findings

As shown in Table 3, the lumbar segment was the most frequently affected site (38.2%), followed by the thoracic (35.7%), lumbosacral (8.0%), thoracolumbar (7.9%), cervical (6.4%), and cervicothoracic spines (3.0%), the sacrococcygeal area being the least frequently involved (0.8%). There was STB in two or more vertebrae in 91.1% of patients, in 67.9% of whom two adjacent vertebrae were involved. Abscesses were detected by CT or MRI in 903 of 1378 patients (65.5%), including paraspinal, prevertebral and psoas abscesses.

### 3.4. Treatment and Prognosis

**Figure 2:**
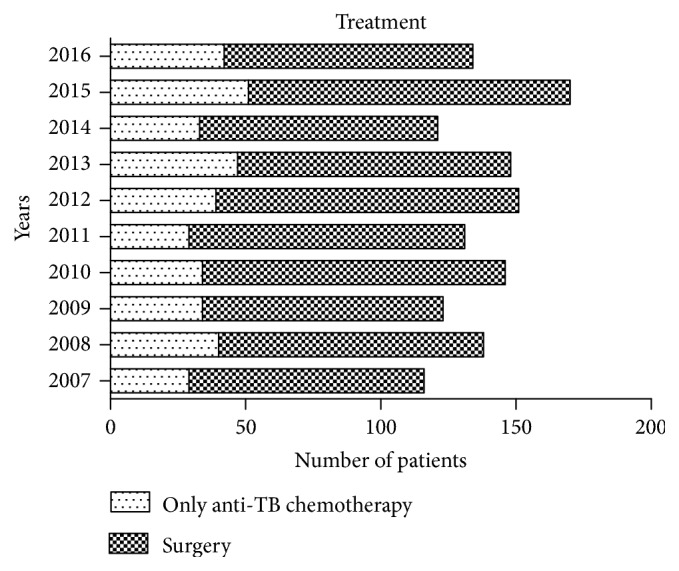
Number of patients treated by surgery or anti-TB chemotherapy only in each year.

As to distribution of treatment (Figure 2), 1000 patients (72.6%) were treated surgically and 378 (27.4%) with anti-TB drugs only. The proportion of patients undergoing surgery did not differ significantly over the 10 years of the study (P > 0.05). The surgical approach (single anterior, single posterior, or combined anterior–posterior approach) was selected on the basis of each patient's condition. Minimally invasive CT-guided percutaneous catheter drainage and focal catheter infusion was used in patients with huge iliopsoas abscesses; these procedures are included in the surgical group.

**Figure 3:**
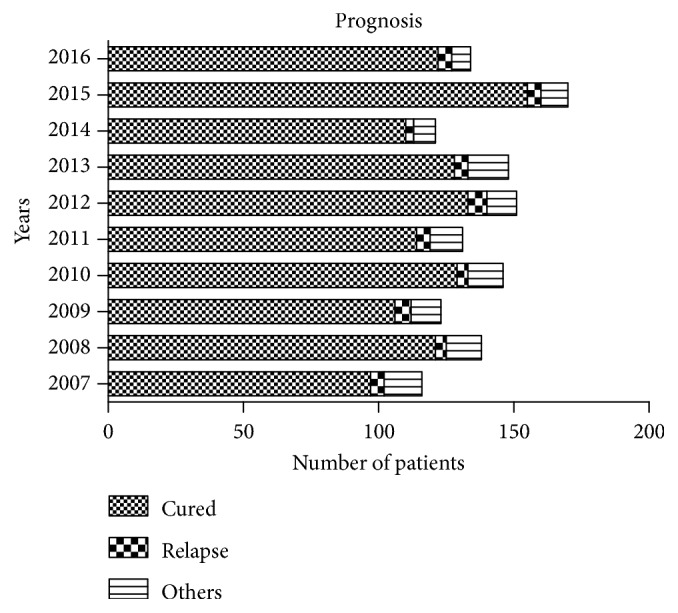
Number of patients with various outcomes in each year.

As to distribution of prognosis (Figure 3), 1215 patients (88.2%) gradually achieved cure within 18 months after hospital admission and initiation of treatment. The 49 patients (3.5%) who relapsed all had postoperative recurrences and required retreatment in hospital. Relapses were presented as postoperative recurrence of TB lesions, abscess formation, sinus formation, progressive kyphosis, or internal fixation fractures, all of which were treated by revision surgery. Follow-up data on the other 114 patients (8.3%) were incomplete. They included patients who were transferred to specialized TB hospitals, those with lost contact, those not cured, and those who died. Poor compliance was the main reason for patients not achieving cure. One death was due to systemic infection.

### 3.5. Comorbidities

**Figure 4:**
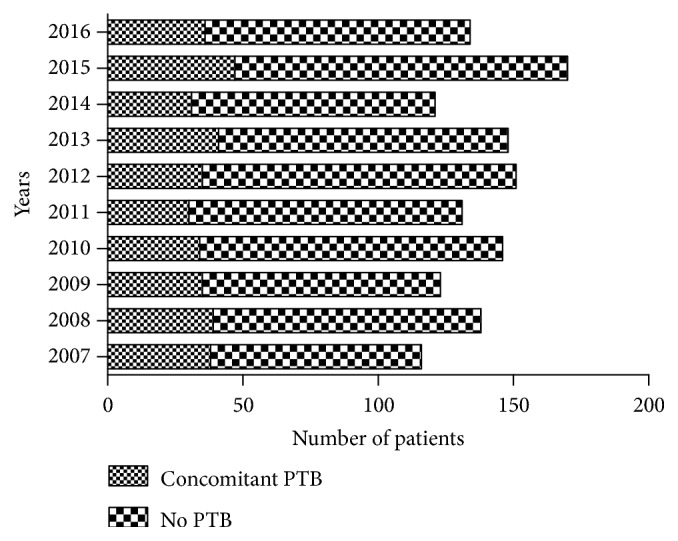
Number of patients with and without concomitant PTB in each year.

**Figure 5:**
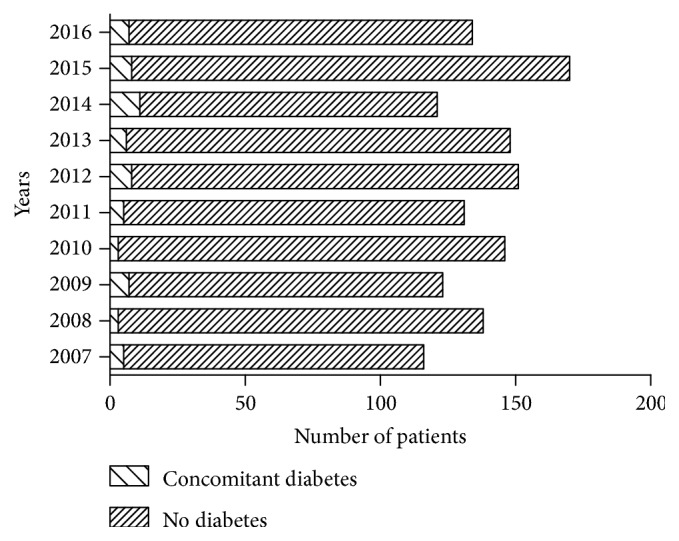
Number of patients with and without concomitant diabetes in each year.

Concomitant PTB, including a previous history of PTB, was present in 366 (26.6%) patients (Figure 4), whereas 63 patients (4.6%) had concomitant diabetes (Figure 5). The proportion of patients with STB accompanied by PTB or diabetes did not differ significantly over the 10 study years (P > 0.05). Among other complications, two patients with thoracolumbar STB had accompanying renal TB and one with cervical STB had accompanying lymph node TB. None of the patients in this study were HIV-positive.

Correlations between various variables and STB and PTB are shown in Table 4. Of the patients with PTB, 63.4% were males, 70.8% had accompanying abscesses, 37.7% had STS, 56.6% had accompanying neurological damage, and 61.2% received non-surgical treatment only. Sex, abscess, STS, neurological injury, and surgery were positively correlated with PTB (P < 0.05). The cure rate did not differ significantly between patients with and without PTB (P > 0.05).

Of the patients with concomitant diabetes, 87.3% were middle-aged or older, 44.4 % had STS, 46.0% required non-surgical treatment only, and the cure rate was 76.2% (Table 5). Age, STS, surgery and prognosis were correlated with the presence of diabetes (P < 0.05).

### 3.6. Comparison of Data according to Time Period

Comparisons of data in the first and second group of 5 years are shown in Table 6. Group A (January 2007–December 2011) comprised 654 patients and Group B (January 2012–December 2016) 724. There was a greater proportion of patients aged over 60 years, urban patients, and medical staff with STB in Group B than in Group A (P < 0.05), the proportion of older patients, urban patients, and medical staff with STB having increased by 6.1%, 5.2%, and 1.3%, respectively.

## 4. Discussion

According to a 2017 WHO report [1], 603,000 male and 292,000 female patients were newly diagnosed with TB in China, this male to female ratio being 2.1:1. In the present study, the male to female ratio of the 1378 patients with STB was 1.4:1, and there was no significant change in that ratio over the 10 years of the study. Young and middle-aged adults (18–60 years old) accounted for 1378 (69.9%) of the patients with STB. This age group provides the main labor force, which is important in that long-term physical labor increases the load on and activity of the spine, resulting in chronic spinal damage, thus increasing susceptibility to MTB. Children had the lowest incidence (7.3%), which may be attributable to the protective effect of BCG inoculation, which is sustained into adolescence [7].

In 2010, the fifth national TB survey showed an increase in the number of older patients with bone and joint TB [8], and in the present study we found that the number of patients aged >60 years increased significantly over time. This increase in older patients may be associated with the aging of the general population [9], lengthening lifespans, poor immunological function, and the increased number of comorbidities in this age group [10]. In addition, STB in older persons is characterized by complexity, disease, slow recovery, resistance to treatment, and atypical symptoms [11]. This trend has hampered developments in public health and social conditions and increased difficulties in prevention and treatment of STB.

Worldwide, 80% of patients with STB are in developing countries and poverty-stricken areas. In China, 80% of patients with STB are in rural areas [8]. In this study, 72.8% patients were from rural areas, which are associated with poor economic level and poor medical and health conditions. The proportion of urban patients with STB increased from 24.5% to 29.7% between the two five-year periods of the present study; this may be related to the increasing proportion of Chinese persons in urban areas. Developments in transportation and increases in population mobility may have contributed to this change. We identified more patients with STB in Changsha, Shaoyang, Yueyang, and Yiyang than in other cities of Hunan province. Possible reasons for this discrepancy may include comprehensive factors affecting the availability of treatment, the population of each city, and variations in economic situation between regions. Thus, screening for STB is recommended for improving prevention, early diagnosis, and treatment of STB in the cities with a high incidence of this condition.

In this study, 66.6% of the patients were farmers and manual workers, who had been engaging in stooped labor, which may increase the prevalence of STB. It is worth noting that the incidence of medical staff increased significantly (P < 0.05) over the 10 years of the study. Medical staff are exposed to patients with diagnosed or suspected TB, which confers a high risk of TB infection [12].

Back pain is the most frequent symptom of STB [13]. Such pain is typically localized to the site of involvement, which is most commonly the thoracic and lumbar spine. In one study, chronic back pain was the only symptom in 61% of patients with STB [3]. In the present study, 92.5% of inpatients had pain at the site of lesion and this was the main reason for patients seeking medical evaluation and treatment. The mechanisms by which STB causes pain are as follows. First, pain may be caused by spinal instability due to destruction of the vertebral body. Second, neuropathic pain may result from inflammatory stimuli associated with TB lesions, such as abscesses and necrotic bone. Radicular pain was present in 40.1% of our patients as a result of inflammatory stimuli of nerve root. STS of TB, indicating presence of active disease, are present in approximately 20%~30% of patients with osteoarticular TB [13]. In this study, 32.1% of patients presented with STS of TB, including low fever, fatigue, emaciation, and night sweats. High fever may also occur during the acute phase of TB. Typical STS of TB can contribute to the diagnosis of STB; however, 67.9% of our patients lacked such symptoms.

STB is prone to cause neurological injury and the rate of disability is high. The incidence of neurological impairment in patients with STB reportedly varies from 23% to 76% [14]. In this study, 49.9% patients had neurologic impairment, 1.5% of them having ASIA Grade A and 2.8% Grade B, which represent severe neurologic damage. Patients with STB and neurologic impairment often need surgical treatment to create space to enable nerve recovery. Impaired neural function can recover to different degrees after surgery; however, recovery in patients with severe neurologic impairment is characteristically poor.

STB often involves two vertebrae because the segmental arteries bifurcate to supply two adjacent vertebrae. Spread of MTB beneath the anterior or posterior longitudinal ligaments can also result in involvement of multiple contiguous vertebrae. In this study, 67.9% of patients had two involved adjacent vertebrae, which comprised one motion segment. The most frequently involved vertebrae were lumbar, followed by thoracic. This localization may be related to the anatomical factors of high level of mobility and load forces in the lumbar region and the long segments in the thoracic region. The incidence was only 0.8% in sacrococcygeal vertebrae, likely because of its short segments, low mobility, and low load forces. Abscess formation around a vertebral lesion, which is not uncommon and prone to extreme enlargement, is another characteristic feature of STB. In this study, 65.5% of patients had abscesses. The 34.5% without abscesses may be more difficult to diagnose. Differential diagnoses include other diseases that cause bone destruction, such as spinal tumors.

Anti-TB drug treatment is the gold standard for the treatment of STB, whereas surgical treatment plays an important role as adjuvant therapy. The aim of surgery is to completely remove a lesion, achieve spinal decompression, reconstruct spinal stability, and restore a normal spinal sequence [15]. In this study, 72.6% of inpatients underwent surgery and their cure rate was as high as 88.2%. The relapse rate was 3.5% (49 patients). Reasons for recurrence may include erratic anti-TB drug treatment, malnutrition, comorbidities, and inadequate rest. The patients with relapses were all cured by revision surgery, including debridement, drainage, and focal catheter infusion with anti-TB drugs.

Five percent of patients with PTB reportedly have accompanying STB [16, 17]. Approximately 33%–50% of patients with STB have an associated primary lung focus or report a history of PTB [18]. In our study, 26.6% of patients with STB had accompanying PTB. We found that concomitant STB and PTB had the following characteristics. This combination was more common in male patients and was associated with abscesses, STS, neurological injury, and non-operative treatment. The influence of smoking, drinking and other lifestyle factors [19] may account for the predominance of PTB in men. Given that the STS of fever and night sweats are commonly present in patients with PTB, it is unsurprising that they are also common in patients with STB and PTB. The probability of abscess and neurological injury may be related to the extent of lesion; the specific reasons need further study. Patients with STB complicated by active PTB are prone to undergo conservative treatment. Surgery may cause spread of MTB.

The prevalence of TB was three to six times higher in patients with diabetes than in those without diabetes; additionally, the incidence of TB was three times higher in patients with poorly controlled diabetes than in those with ideally controlled diabetes [20]. Active TB, being an infection, can aggravate diabetes and may even induce acute ketoacidosis. Patients with diabetes and TB usually have the characteristics of long course, severe illness, rapid progression, and slow rehabilitation, which make treatment difficult [21, 22]. Moreover, patients with diabetes and STB have impaired anti-infection and tissue repair ability. Internal fixation and implants [23] are often needed for STB surgery. Active infection often exacerbates the patient's condition and affects the prognosis, sometimes leading to failure of surgery [24]. In our study, 63 patients (4.6%) had accompanying diabetes. We found that patients with STB and diabetes had the following characteristics. They were more commonly aged over 45 years and were prone to have STS and undergo non-surgical treatment. In addition, their prognosis was relatively poor. If surgery is needed in these patients, attention should be paid to the following: infection, wound healing [25], osteoporosis [26], stability of internal fixation [27] and vascular lesions [28].

The following limitations of this study are worth noting. First, the number of patients was relatively small and they were all inpatients. Because data on them is inadequate and difficult to obtain, outpatients were not included in this study; this may have resulted in a bias in outcomes, such as the higher rate of surgery and no HIV/AIDS case. Second, results of tests for drug resistance were unavailable.

## 5. Conclusions

STB remains a severe problem endangering the public health and cannot be ignored. Most patients ignore its symptoms and therefore receive untimely treatment. Thus, surveillance for and treatment of STB should be strengthened in south-central China. In addition to the existing database for PTB, a database of patients with STB should be set up throughout China.

## Figures and Tables

**Table 1:** Demographics and epidemiology of inpatients with STB.

Year	Gender		Age (yeas)				Residence		Occupation				Total
	Male	Female	<18	18~45	46~60	>60	Rural	Urban	Farmer	Worker	Student	Others	
2007	70	46	7	53	42	14	84	32	59	24	10	23	116
2008	82	56	11	58	41	28	96	42	67	30	12	29	138
2009	65	58	15	52	33	23	91	32	65	22	16	20	123
2010	86	60	10	66	38	32	122	24	75	24	14	33	146
2011	75	56	6	61	33	31	101	30	71	21	9	30	131
2012	91	60	10	71	31	39	115	36	69	30	13	39	151
2013	79	69	13	59	43	33	99	49	65	28	14	41	148
2014	75	46	6	42	47	26	76	45	56	21	7	47	121
2015	101	69	11	63	46	50	122	48	79	27	17	47	170
2016	81	53	11	36	49	38	97	37	53	32	16	33	134

Total	805	573	100	561	403	314	1003	375	659	259	128	332	1378
Rate (%)	58.4	41.6	7.3	40.7	29.2	22.8	72.8	27.2	47.8	18.8	9.3	24.1	
*χ*2	4.583		46.592				20.252		30.756				
P	0.869		0.011				0.016		0.281				

**Table 2:** Clinical presentation of inpatients with STB.

Year	Back pain		Radiating pain		Systemic toxicity symptoms		Neurological injury				
							(ASIA grade)				
	Yes	No	Yes	No	Yes	No	A	B	C	D	E
2007	108	8	55	61	38	78	2	3	6	50	55
2008	127	11	60	78	47	91	1	2	10	58	67
2009	109	14	49	74	39	84	2	2	14	40	65
2010	134	12	51	95	44	102	1	3	13	64	65
2011	118	13	49	82	47	84	3	2	13	50	63
2012	142	9	52	99	66	85	4	5	12	52	78
2013	139	9	59	89	37	111	2	6	8	61	71
2014	116	5	56	65	29	92	1	3	11	33	73
2015	154	16	56	114	52	118	2	6	9	52	101
2016	128	6	65	69	44	90	3	6	15	57	53

Total	1275	103	552	826	443	935	21	38	111	517	691
Rate (%)	92.5	7.5	40.1	59.9	32.1	67.9	1.5	2.8	8.1	37.5	50.1
*χ*2	9.607		16.782		18.017		26.902				
P	0.383		0.052		0.035		0.081				

**Table 3:** Imaging findings of inpatients with STB.

Year	Lesion sites							Involved number of vertebrae			Abscess	
	C	CT	T	TL	L	LS	S	1	2	≧3	With	Without
2007	6	5	53	7	39	6	0	10	81	25	75	41
2008	8	8	59	11	40	11	1	16	92	30	87	51
2009	5	4	49	16	39	9	1	12	86	25	80	43
2010	11	3	59	12	47	13	1	17	104	25	98	48
2011	2	7	52	12	47	9	2	6	85	40	89	42
2012	8	1	55	10	60	14	3	13	101	37	109	42
2013	11	3	49	9	68	8	0	14	97	37	90	58
2014	12	2	28	14	52	12	1	10	79	32	81	40
2015	12	2	53	10	80	11	2	15	124	31	112	58
2016	13	6	35	8	55	17	0	10	86	38	82	52

Total	88	41	492	109	527	110	11	123	935	320	903	475
Rate (%)	6.4	3.0	35.7	7.9	38.2	8.0	0.8	8.9	67.9	23.2	65.5	34.5
*χ*2	81.027							17.655			6.582	
P	0.01							0.479			0.681	

C: Cervical; CT: Cervicothoracic; T: Thoracic; TL: Thoracolumbar; L: Lumbar; LS: Lumbosacral; S: Sacrococcygeal.

**Table 4:** Correlation analysis between STB and PTB.

Variate	Type	Without PTB		With PTB		Total	*χ*2	P
		N	%	N	%			
Gender	Male	573	56.6	232	63.4	805	5.068	0.024
	Female	439	43.4	134	36.6	573		
Abscess	Yes	644	63.6	259	70.8	903	6.047	0.014
	No	368	36.4	107	29.2	475		
Surgery	Yes	776	76.7	224	61.2	1000	32.347	0.000
	No	236	23.3	142	38.8	378		
Systemic toxicity symptoms	Yes	305	30.1	138	37.7	443	7.055	0.008
	No	707	69.9	228	62.3	935		
Neurological injury	Yes	480	47.4	207	56.6	687	8.955	0.003
	No	532	52.6	159	43.4	691		
Prognosis	Cure	891	88.0	319	87.2	1214	0.490	0.484
	Others	121	12.0	47	12.8	163		

**Table 5:** Correlation analysis between STB and diabetes.

Variate	Type	Without diabetes		With diabetes		Total	*χ*2	P
		N	%	N	%			
Age (yeas)	>45	662	50.3	55	87.3	717	32.904	0.000
	≦45	653	49.7	8	12.7	661		
Systemic toxicity symptoms	Yes	415	31.6	28	44.4	443	4.576	0.032
	No	900	68.4	35	55.6	935		
Surgery	Yes	966	73.4	34	54.0	1000	11.474	0.001
	No	349	26.6	29	46.0	378		
Prognosis	Cure	1167	88.7	48	76.2	1215	9.086	0.003
	Others	148	11.3	15	23.8	163		

**Table 6:** Comparison of 10 years' data.

Variate	Type	Group A		Group B		Total	*χ*2	P
		(2007-2011)		(2012-2016)				
		N	%	N	%			
Gender	Male	378	57.8	427	59	805	0.197	0.657
	Female	276	42.2	297	41	573		
Age (yeas)	≦60	526	80.4	538	74.3	1064	7.312	0.007
	>60	128	19.6	186	25.7	314		
Residence	Rural	494	75.5	509	70.3	1003	4.747	0.029
	Urban	160	24.5	215	29.7	375		
Occupation	Medical staff	6	0.9	16	2.2	22	4.154	0.046
	Others	648	99.1	708	97.8	1356		
Back pain	Yes	596	91.1	679	93.8	1275	3.497	0.061
	No	58	8.9	45	6.2	103		
Radicular pain	Yes	264	40.4	288	39.6	552	0.049	0.825
	No	390	59.6	436	60.4	826		
Systemic toxicity symptoms	Yes	215	32.9	228	31.5	443	0.301	0.583
	No	439	67.1	496	68.5	935		
Abscess	Yes	429	65.6	474	65.5	903	0.002	0.964
	No	225	34.4	250	34.5	475		
Neurological injury	Yes	339	51.8	348	48.1	687	1.952	0.162
	No	315	48.2	376	51.9	691		
Involved number of vertebrae	<3	509	77.8	549	75.9	1058	0.771	0.380
	≧3	145	22.2	175	24.1	320		
Surgery	Yes	488	68.5	512	70.7	1000	2.625	0.105
	No	166	31.5	212	29.3	378		
PTB	Yes	176	26.9	190	26.2	366	0.079	0.779
	No	478	73.1	534	73.8	1012		
Diabetes	Yes	23	3.5	40	5.5	63	3.176	0.075
	No	631	96.5	684	94.5	1315		
Prognosis	Cure	567	86.7	648	89.5	1215	2.593	0.107
	Others	87	13.3	76	11.9	163		

## Data Availability

The data used to support the findings of this study are included within the article.
